# Visual Field Improvement and Electrode Extrusion Prevention by Extended Endaural Incision With Cavity Obliteration for Cochlear Implantation in Ears After Canal Wall-Down Mastoidectomy: Experience of Four Cases

**DOI:** 10.7759/cureus.54570

**Published:** 2024-02-20

**Authors:** Kohei Fukuda, Keiji Tabuchi, Yuki Hirose, Shin Matsumoto, Masahiro Adachi

**Affiliations:** 1 Otolaryngology - Head and Neck Surgery, University of Tsukuba, Tsukuba, JPN

**Keywords:** ear surgery, surgical techniques, extended endaural incision, canal wall down mastoidectomy, cochlear implantation

## Abstract

There is no global consensus on the surgical technique of cochlear implantation (CI) in ears with an open cavity after canal wall-down (CWD) mastoidectomy. Here, we report CI surgery with an endaural incision for the ears after CWD mastoidectomy. The endaural incision was extended upward to obliterate the open cavity of the temporal fascial flap. The endaural incision was extended downward to close the open cavity inlet. After inserting the implanted electrode, the open cavity was obliterated using a temporal fascial flap, and the cavity was closed at the inlet. We performed this type of CI surgery in four ears in three patients. This extended endaural incision provided an excellent view for pedicling the temporal fascial flap with the superficial temporal artery and for open cavity closure without any serious complications. This technique allowed us to opt for CI surgery of the ears after CWD mastoidectomy.

## Introduction

Cochlear implantation (CI) is a remarkably effective treatment for patients with sensorineural hearing loss. In recent years, the number of CI surgeries performed has increased in Japan [1]. Globally, CI is recognized as the world’s most successful sensory prostheses, restoring hearing for over 800,000 individuals worldwide, with the majority experiencing enhanced speech perception. It is considered the gold standard for treating severe to profound bilateral sensorineural hearing loss, particularly in advanced economies [2]. Generally, the electrode of the CI is inserted into the cochlea after canal wall-up mastoidectomy and posterior tympanotomy. However, CI is occasionally conducted in patients who have undergone canal wall-down (CWD) mastoidectomy (the posterior wall of the external auditory canal was removed due to previous middle ear diseases such as cholesteatoma). There is no global consensus on the management of open cavities during CI surgery. If an external ear canal wall is newly created and the electrode is inserted through the newly created mastoid, postsurgical electrode extrusion into the external auditory canal can be a problem. Several studies have recommended removing the skin of the open cavity and closing its inlet, a technique known as blind sac closure [3]. The obliteration of the cavity has also been reported. Rambo et al. (1958) used a pedicled temporal fascial flap for cavity obliteration [4]. Here, we report an extended endaural (just anterior to the auricle) incision to perform CIs in ears after CWD mastoidectomy. The open cavities were obliterated using pedicled temporal fascial flaps. This incision provides a good view to create a temporal fascial flap, including its pedicle, and to close the open cavity. We believe that with this surgical technique, we can achieve a resilient defense against infection and prevent electrode extrusion. Furthermore, an operated ear with this technique does not need frequent outpatient visits for ear cleaning.

## Technical report

Methodology

**Figure 1:**
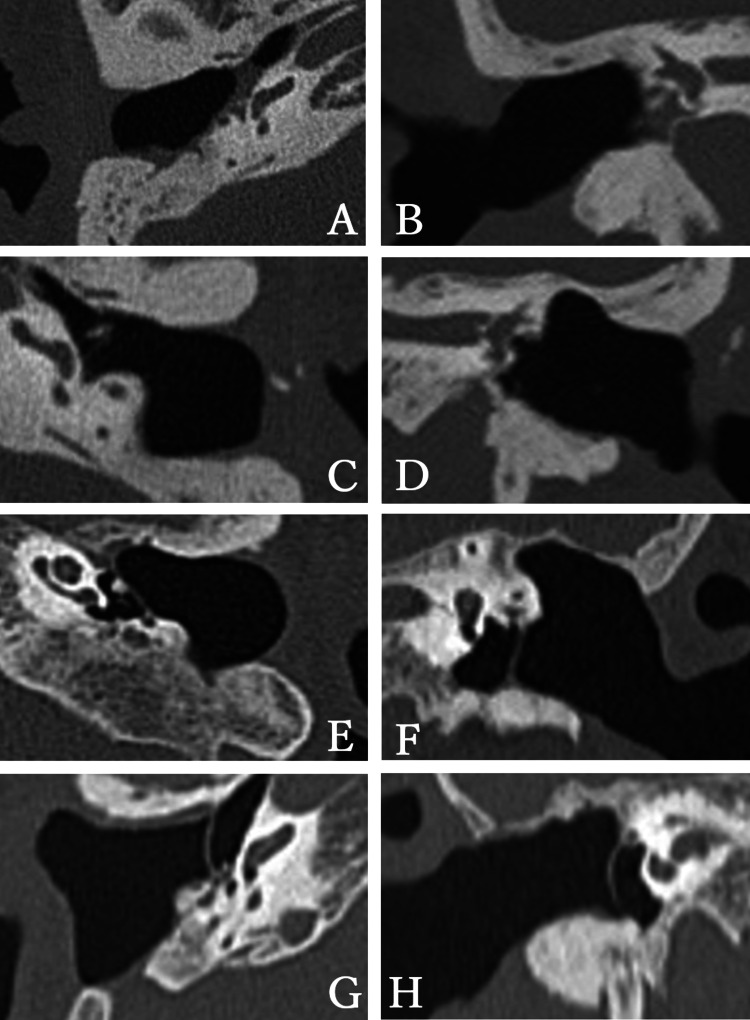
CT scan of cases 1, 2, and 3. (A-D) Preoperative temporal bone CT of case 1. The right ear (A: axial, B: coronal). The left ear (C: axial, D: coronal). (E, F) Preoperative temporal bone CT in case 2. Left ear (E: axial, F: coronal). (G, H) Preoperative temporal bone CT in case 3. Right ear (G: axial, H: coronal).

Inclusion criteria were patients with CWD who underwent cochlear implant surgery at our institution between October 2020 and December 2022. All patients were limited to those with severe to profound sensorineural hearing loss, making conversation challenging with conventional hearing aids. Individuals with current or recurrent otitis media and those suspected of recurrent cholesteatoma were excluded from the study. Patients with bilateral aplasia of the vestibulocochlear nerve were also excluded. All patients underwent thorough preoperative examinations, including ear examination, CT, MRI, pure-tone audiometry, speech audiometry, auditory brainstem response, chest X-ray, and electrocardiogram, to ensure their suitability for surgery and safety. CI surgery with this technique was performed in four ears of three patients. A 79-year-old man (case 1), a 68-year-old woman (case 2), and an 82-year-old woman (case 3) had undergone bilateral CWD mastoidectomy for cholesteatoma and had clean open cavities after CWD surgery. The CT scans of cases 1, 2, and 3 are shown in Figure 1. All patients exhibited severe bilateral sensorineural hearing loss. Depending on their preferences, cases 1, 2, and 3 received bilateral, left, and right CIs, respectively. A profile of the patients is presented in Table 1.

**Table 1:** Characteristics of the patients. CWD: canal wall down

Case	Age, sex	Side	Reason for CWD	Open cavity health
1	79, male	Right	Cholesteatoma	Good. No infection and no recurrence
1	79, male	Left	Cholesteatoma	Good. No infection and no recurrence
2	68, female	Left	Chronic otitis media	Good. No infection
3	82, female	Right	Chronic otitis media	Good. No infection

The extended endaural incision for the pedicled temporal fascial flap and the closure of the open cavity inlet

**Figure 2:**
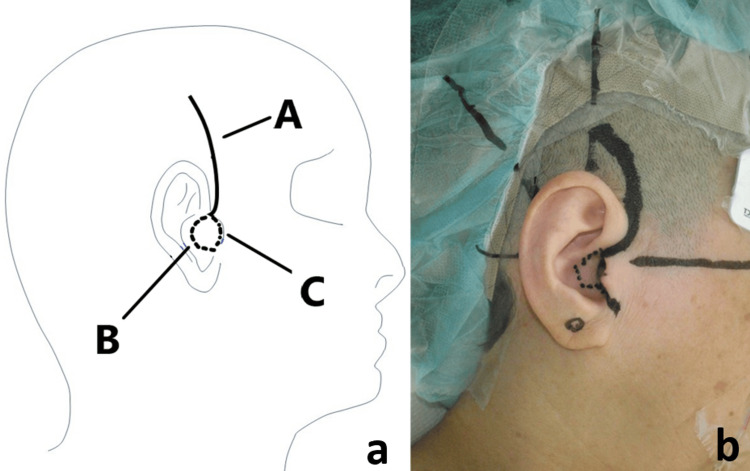
Extended endaural incision (case 1). (a) Line A: Endaural incision is extended upward. This allows to harvest a sufficient amount of anteroinferior pedicled temporal fascial flap while keeping the superficial temporal artery in view. Line B: The incision is extended to the tip of the tragus. The skin of the external auditory canal (EAC) side of the tragus is removed with the skin of the open cavity. Line C: The incision is also extended on the cavum conchae. The skin of the cavum conchae should also be removed in addition to the skin of the open cavity. (b) Photograph of the incision line: The incision line of the cavum conchae is represented by the dotted line.

An endaural incision was made and extended upward, as illustrated by line A in Figure 2. The skin flap was elevated from the temporal fascia to provide a surgical view of the pedicled temporal fascial flap. The periosteum of the temporal region was elevated from the temporal bone to create the pocket (bed) for the CI body between the periosteum and bone. The incision was extended downward to the cavum conchae and tragus to close the open cavity inlet (Figure 2, lines B and C). Subsequently, all the skin covering the open cavity, including the tympanic membrane, external auditory canal, and cavum conchae, was completely removed. The tympanic orifice of the eustachian tube was closed with muscle or fascia to prevent the entry of infectious agents from the nasopharynx.

**Figure 3:**
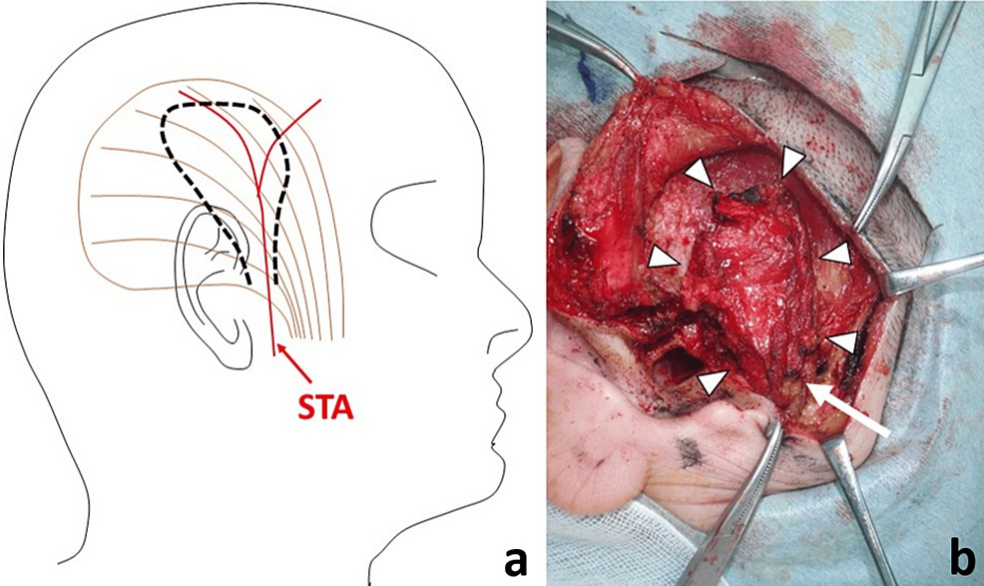
Temporal fascial flap (case 1). (a) Schematic view: The main flow is supplied by the superficial temporal artery (STA). The dotted line is designed as the anteroinferior pedicled temporal muscle flap receiving the abundant blood flow of STA. (b) Photograph: The flap is made (arrowhead) with STA (arrow).

As shown in Figure 3, a temporal fascial flap was created. The fascial flap was anteroinferiorly stemmed to ensure that it received an abundant blood flow from the superficial temporal artery (STA).

Electrode insertion and wound closure

For the cochlear implant, the rim of the round window niche was removed using a small diamond bur. After the round window membrane was incised with a needle, the electrode array was inserted into the cochlea. The temporal fascial flap was then rotated into the open cavity to cover the electrode and obliterate the cavity. The CI receiver was inserted into the space at the superior-posterior bony wall of the open cavity, between the bone and the periosteum, without subsequent fixation. For wound closure, the tragal cartilage was rotated over the denuded cavum conchae and sutured to the conchae, thus closing the open cavity inlet.

Comparative analysis of operation time

We compared the operative time between the current surgical technique and conventional CI procedures performed by the same surgeon during the same period. During the same period, a single surgeon performed conventional unilateral cochlear implant surgeries on 35 ears. In cases of bilateral simultaneous surgeries, the total operative time was evenly divided by two to determine the duration for each ear.

Results

The patients, surgeries, and outcomes are summarized in Table 2. CI with an extended endaural incision provides a good view for the temporal fascial flap. It provides a particularly excellent surgical view of the STA. This approach also provides good access to the round window. After drilling the bone of the rim of the round window niche, all electrodes (CI632, Cochlear, Sidney, Australia) were smoothly inserted into the cochlea. The total operation time was 278 minutes for both ears in case 1, 78 minutes for the left ear in case 2, and 136 minutes for the right ear in case 3. The average duration was 123 minutes per ear. During the same period, a single surgeon performed routine unilateral cochlear implant surgeries on 35 ears, with an average operation time of 75.5 ± 31.6 minutes (adjusted for bilateral cases by halving the operation time). A significant difference in operation times was observed between the two groups (Mann-Whitney U test, p-value = 0.017). Two of the four ears exhibited a mild postoperative hematoma at the cephalic side of the auricle that spontaneously resolved in a week.

**Table 2:** Case summary (age, sex, advantage, operation time, complications, follow-up periods, and other complications). STA: superficial temporal artery

Case	Age, sex	Side	STA view	Fascial flap view	Round window view	Electrode insertion	Operation time (minute)	Postoperative complications	Follow-up periods (month)	Complications other than postoperative complications
1	79, male	Right	Good	Good	Good	Round window	278 for both sides	Mild hematoma at the cephalic side of the auricle	37	None
1	79, male	Left	Good	Good	Good	Round window	278 for both sides	None	37	None
2	68, female	Left	Good	Good	Good	Round window	78	Mild hematoma at the cephalic side of the auricle	36	None
3	82, female	Right	Good	Good	Good	Round window	136	None	12	None

## Discussion

Although CI is generally performed in the healthy temporal bone, there are cases in which cochlear implants must be placed in the open mastoid cavity because of previous middle ear diseases. In such cases, the treatment of the open cavity varies depending on the patient’s condition and the surgeon’s preference.

There are two main ways to treat an open cavity, namely, (1) partially obliterating the open cavity or (2) closing the open cavity [5]. The advantage of partial obliteration is that it is easy to identify inflammatory middle ear diseases through the tympanic membrane after CI because the tympanic membrane remains in the post-CI ear. Conversely, the disadvantage of this method is the possibility of persistent infection through the external auditory canal and electrode array extrusion. In the case of external auditory canal closure, the advantage is that the frequency of infection and electrode array extrusion is less than that associated with previous methods [6,7]. Another advantage is that it does not necessitate swimming restrictions. However, it is difficult to identify inflammatory middle ear diseases because the tympanic membrane is removed during surgery. Moreover, thorough removal of the squamous epithelium is required because residual squamous epithelium generates cholesteatomas.

Hunter et al. (2016) reported that the rates of surgical complications following CI in CWD mastoidectomy associated with external auditory canal closure and residual open cavities were 14.6% and 30.0%, respectively [3]. Considering these data, we chose to close the open cavity.

Various materials are available for filling an open mastoid cavity, including the temporal fascia and autologous transplantation of abdominal fat and bone pate. In a study by Polo et al. (2016), which reported 110 cases of CI following subtotal petrosectomy [8], abdominal fat grafting was employed in five cases, resulting in electrode extrusion in all five instances. The temporal muscle has also been reported as a material for cavity obliteration [9]. A potential drawback associated with the use of temporal fascia and muscle is the challenge of identifying acquired cholesteatoma through CT imaging. This difficulty arises due to the similarity in CT density between flaps, scars, and cholesteatoma. Moreover, the presence of post-CI artifacts further complicates the accurate depiction of cholesteatoma lesions on MRI. After CI, artifacts are so strong that it is almost impossible to evaluate middle ear lesions on MRI. In this regard, the use of fat appears to have advantages in identifying recurrent cholesteatoma or infection on CT imaging. However, we believe that infection control and prevention of electrode extrusion are top priorities. In this regard, the pedicled temporal fascial flap is a good material because of its abundant blood flow. Considering this disadvantage, it is necessary to thoroughly remove the squamous epithelium of the cavity. Additionally, it is necessary to pay particular attention to the increase in soft tissue density on CT scans and the progression of bone destruction for at least five years [10]. In this study, the observation periods were relatively short (12 months, 36 months, and 37 months, respectively), leaving a potential risk for the occurrence of cholesteatoma.

Postoperative hematoma was the noted complication of the CI surgery. We consider that it may be beneficial to place a penrose drain to prevent this complication. In addition, meticulous hemostasis of the branches of the superficial temporal artery and vein during flap creation is believed to decrease the risk of hematoma formation.

The method of filling the open cavity with pedicled temporal muscle was first reported by Rambo in 1958 [4], who closed the external auditory canal by dissecting and suturing its skin. Blind sac closure is a well-known technique used to close the external auditory canal and is usually adapted for retroaural (just posterior to the auricle) incisions [11]. The retroaural incision is considered superior to the endaural incision in terms of cosmetic outcomes. However, the temporal fascia of the retroaural area was often removed in previous surgeries. The endaural incision significantly improved the field of view for STA and the remaining temporal fascia. In addition, using an endaural incision, it is easy to close the external auditory canal using the tragal cartilage. The tragus was sewn into the cavum conchae, and the rotated tragal cartilage provided a rigid wall. This closure of the external auditory canal with cartilage is considered an effective method that can prevent both electrode extrusion and disruption of external auditory canal closure. The extended endaural incision technique seems reasonable for achieving a good field of view for both temporal fascial harvesting and external auditory canal closure via the tragus.

The introduction of this surgical technique resulted in an average extension of 47.5 minutes in the operation time. The extension in time can be expected to decrease with the proficiency gained in the surgical technique, leading to a reduction in overall operation time.

Limitations of this study include a small sample size and a short observation period. Future efforts should involve accumulating more cases, collecting data on complication rates through long-term observation, and comparing outcomes with other surgical techniques. While the study focused on patients who had already undergone CWD mastoidectomy, future considerations could explore the applicability of this technique in cases with middle ear anomalies or compromised temporal bone development. Validation of the method’s safety and absence of complications will necessitate a larger sample size and an extended follow-up period.

## Conclusions

In this study, we performed CI for the open cavity after CWD mastoidectomy using an extended endaural incision. This surgical method of filling the open cavity with the anteroinferior pedicled temporal fascial flap and closing the cavity using the tragus may be an option for CI in an open mastoid cavity. This method is not entirely new or difficult but is easy and practical for everyone to adopt. In addition, closing the open cavity eliminates the need for outpatient visits for periodic ear cleaning. The operation time was extended by an average of approximately 47 minutes compared to standard cochlear implant surgery, but it was safely completed in all cases. Complications were minimal, with only two out of four cases experiencing mild hematomas that did not require specific interventions. Patients not recommended for this technique include those with infected or recurrent ears. Limitations of this study include a small sample size and a short observation period. Future efforts should involve accumulating more cases, collecting data on complication rates through long-term observation, and comparing outcomes with other surgical techniques. While the study focused on patients who had already undergone CWD mastoidectomy, future considerations could explore the applicability of this technique in cases with middle ear anomalies or compromised temporal bone development. Validation of the method’s safety and absence of complications will necessitate a larger sample size and an extended follow-up period. However, we remain optimistic that this approach has the potential to encourage individuals with severe sensorineural hearing loss, who routinely seek open cavity cleaning from otolaryngologists or their attending physicians, to more actively contemplate cochlear implant surgery.
